# DOES HEARING LOSS MODERATE THE RELATIONSHIP BETWEEN COGNITION AND DEPRESSION?

**DOI:** 10.1093/geroni/igad104.2436

**Published:** 2023-12-21

**Authors:** Jennifer Lister, Aryn Harrison Bush, Jennifer O’Brien, Laura Conover, Nasreen Sadeq

**Affiliations:** University of South Florida, Tampa, Florida, United States; University of South Florida, Tampa, Florida, United States; University of South Florida, Tampa, Florida, United States; University of South Florida, Tampa, Florida, United States; University of South Florida, Tampa, Florida, United States

## Abstract

Hearing loss, depression, and cognitive impairment are common, co-occurring conditions experienced by older adults, but the nature of the relationship among these conditions remains unclear. Both depression and hearing loss have been identified as risk factors for dementia, but for different stages of life (e.g., Singh-Manoux et al., 2017; Livingston et al., 2020). Hearing loss is described as the strongest modifiable risk factor in mid-life, and depression is second only to smoking as the strongest modifiable risk factor in late-life (Livingston et al., 2020). The association between hearing loss and depression is well documented in the literature (e.g., Lawrence et al., 2015). It is possible that hearing loss, as an earlier risk factor, moderates the relationship between depression and cognition. Therefore, as part of an ongoing longitudinal study of early indicators of cognitive decline, we examined the moderating effect of two measures of hearing (pure-tone hearing threshold average, Dichotic Sentence Identification test) on the relationship between depression (Geriatric Depression Scale) and cognition (Montreal Cognitive Assessment, Digit Symbol Substitution test) for a group of 65 older adults (mean age 74). Although none of the hearing or cognitive measures were significant predictors of depression scores on their own, there was a significant negative interaction between the Montreal Cognitive Assessment and pure-tone hearing threshold average, p = 0.048. These results may be interpreted to suggest that individuals with higher (poorer) hearing thresholds show a decrease in depression as their cognitive scores improve, whereas individuals with lower (better) hearing thresholds show the opposite pattern.

